# The role of FoxP3+ regulatory T cells and IDO+ immune and tumor cells in malignant melanoma – an immunohistochemical study

**DOI:** 10.1186/s12885-021-08385-4

**Published:** 2021-05-29

**Authors:** Satu Salmi, Anton Lin, Benjamin Hirschovits-Gerz, Mari Valkonen, Niina Aaltonen, Reijo Sironen, Hanna Siiskonen, Sanna Pasonen-Seppänen

**Affiliations:** 1 Institute of Biomedicine, University of Eastern Finland, P.O. Box 1627 70211, Kuopio campus, Kuopio, Finland; 2 Institute of Clinical Medicine/ Clinical Pathology, University of Eastern Finland, 70029 Kuopio, Finland; 3 Department of Clinical Pathology, Kuopio University Hospital, 70029 Kuopio, Finland; 4 Department of Dermatology, Kuopio University Hospital and University of Eastern Finland, 70029 Kuopio, Finland

**Keywords:** Melanoma, TME, Immunosuppression, Regulatory T cells, FoxP3, IDO

## Abstract

**Background:**

FoxP3+ Regulatory T cells (Tregs) and indoleamine-2,3-dioxygenase (IDO) participate in the formation of an immunosuppressive tumor microenvironment (TME) in malignant cutaneous melanoma (CM). Recent studies have reported that IDO expression correlates with poor prognosis and greater Breslow’s depth, but results concerning the role of FoxP3+ Tregs in CM have been controversial. Furthermore, the correlation between IDO and Tregs has not been substantially studied in CM, although IDO is known to be an important regulator of Tregs activity.

**Methods:**

We investigated the associations of FoxP3+ Tregs, IDO+ tumor cells and IDO+ stromal immune cells with tumor stage, prognostic factors and survival in CM. FoxP3 and IDO were immunohistochemically stained from 29 benign and 29 dysplastic nevi, 18 in situ -melanomas, 48 superficial and 62 deep melanomas and 67 lymph node metastases (LNMs) of CM. The number of FoxP3+ Tregs and IDO+ stromal immune cells, and the coverage and intensity of IDO+ tumor cells were analysed.

**Results:**

The number of FoxP3+ Tregs and IDO+ stromal immune cells were significantly higher in malignant melanomas compared with benign lesions. The increased expression of IDO in melanoma cells was associated with poor prognostic factors, such as recurrence, nodular growth pattern and increased mitotic count. Furthermore, the expression of IDO in melanoma cells was associated with reduced recurrence˗free survival. We further showed that there was a positive correlation between IDO+ tumor cells and FoxP3+ Tregs.

**Conclusions:**

These results indicate that IDO is strongly involved in melanoma progression. FoxP3+ Tregs also seems to contribute to the immunosuppressive TME in CM, but their significance in melanoma progression remains unclear. The positive association of FoxP3+ Tregs with IDO+ melanoma cells, but not with IDO+ stromal immune cells, indicates a complex interaction between IDO and Tregs in CM, which demands further studies.

**Supplementary Information:**

The online version contains supplementary material available at 10.1186/s12885-021-08385-4.

## Background

The incidence of malignant cutaneous melanoma (CM) has been increasing rapidly for the past decade, and the increase is estimated to continue [1]. Although CM is a highly immunogenic cancer, it can evade the immune system by forming an immunosuppressive tumor microenvironment (TME). An understanding of the role of immune escape has led to novel immunotherapies to treat the metastatic disease [2]. Immune evasion results from several factors that weaken the effect of melanoma-specific effector T cells. These factors include anti-inflammatory cytokines, such as IL-10, defects in antigen presentation of melanoma cells and expression of immune checkpoint molecules, such as PD-L1 and CTLA-4. Furthermore, tumor immunosuppression results from the presence of immunosuppressive cells, such as tumor-associated macrophages (TAMs) and regulatory T cells (Tregs), as well as cells that express amino-acid catabolizing enzymes like indoleamine-2,3-dioxygenase (IDO) [3].

Regulatory T cells (Tregs) are a highly immunosuppressive subpopulation of CD4+ T cells expressing Forkhead box P3-transcription factor (FoxP3). Tregs help to maintain self-tolerance and immune homeostasis in healthy individuals and they play a crucial role in preventing autoimmune responses. In cancer, Tregs silence the anti-tumor immune effect and thus promote tumor growth by suppressing the proliferation and cytokine secretion of effector T lymphocytes. Tregs act by secreting inhibitory cytokines, such as IL-10 and TGF-β, expressing immune checkpoint molecule CTLA-4, degrading ATP, destroying effector T cells through direct cytotoxicity by secretion of granzyme and perforin, and inhibiting effector T cell differentiation by consuming IL-2, through expression of CD25 [4].

Indoleamine-2,3-dioxygenase (IDO) is a cytosolic enzyme that catalyses the first and rate-limiting step of tryptophan catabolism by converting tryptophan to kynurenine. Tryptophan depletion is an immunomodulatory process because, the decrease in tryptophan inhibits effector T cell proliferation and activates apoptosis in effector T cells, and an increase in kynurenine and its downstream metabolites promote Treg differentiation [5]. IDO has both immunogenic and immunosuppressive effects, which are controlled by local factors such as IL-6, IL-12, IL-10, IFN-γ, CTLA-4 and PD-1. These factors modulate IDO expression and help to maintain immune homeostasis. In addition to effector T cell inhibition and Treg activation, the immunosuppressive effects of IDO include natural killer cell (NK) inhibition, as well as activation of dendritic cells (DCs) and myeloid-derived suppressor cells (MDSCs). In the early elimination phase of cancer progression, IDO is produced at low levels within the TME and it inhibits tumor growth. In the immune escape phase, IDO production is increased, leading to immunosuppressive effects and tumor progression [6]. In TME, IDO-expressing cells include DCs, macrophages, MDSCs [6] and some cancer cells, including gastric, colon and renal cell carcinomas [7].

Tregs and IDO are known to be part of the immunosuppressive TME in melanoma [2]. However, there are controversial results regarding the association between FoxP3+ Tregs and tumor stage and survival in CM. Most studies have showed that high amounts of IDO+ stromal immune cells and IDO+ melanoma cells associate with a poor prognosis in CM. However, the correlation between IDO and FoxP3+ Tregs has not been thoroughly studied, even though it is known that IDO is an important regulator of Tregs activity [8].

In the present study, we investigated the associations of FoxP3+ Tregs and IDO+ stromal immune and tumor cells with prognostic factors and survival in CM. In addition, the correlation between FoxP3+ Tregs and IDO+ stromal immune and IDO+ tumor cells was examined. Our objective was to enhance our understanding of the role and interrelationship of these important immunosuppressive factors of the TME in melanoma progression. Our results show that the number of FoxP3+ Tregs and IDO+ stromal immune cells is significantly higher in malignant lesions, compared with benign nevi. Increased numbers of IDO-expressing tumor cells was associated with poor prognostic factors. Our data also shows a positive correlation between IDO+ tumor cells and FoxP3+ Tregs, but the number of FoxP3+ Tregs was not associated with tumor stage or survival.

## Methods

### Histological specimens

This retrospective study consists of 29 benign nevi (5 junctional nevi, 14 intradermal nevi, 10 composite nevi), 29 dysplastic nevi, 18 in situ melanomas, 48 superficial melanomas (Breslow depth < 1 mm), 62 deep melanomas (Breslow depth > 4 mm) and 67 lymph node metastases. The samples were histopathologically diagnosed between the years 1980 and 2010 in Kuopio University Hospital. Only pT1 and pT4 melanomas were included in the study, as these tumors have clearly distinct prognosis.

From a total of 253 samples, 240 were stained for FoxP3 and 252 for IDO. Highly pigmented samples, as well as samples with destroyed tissue structure or large necrotic areas were omitted, yielding 183 representative samples for FoxP3 analysis and 193 for IDO analysis (Table 1). Analysed samples including clinical data are listed in Table 2. These sample sizes are sufficient to observe correlations or differences between groups. Out of total of 101 samples with clinicopathological data, data were missing from 0 to 3 samples depending on variable. Histopathological parameters for the samples were provided by an expert pathologist (RS).

**Table 1:** Numbers of FoxP3 and IDO stainings evaluated by hotspot method

Sample sizes	FoxP3 stainings	IDO stainings
Benign nevi	25 (14%)	26 (13%)
Dysplastic nevi	27 (15%)	13 (7%)
In situ melanoma	15 (8%)	46 (24%)
pT1	36 (20%)	35 (18%)
pT4	39 (21%)	49 (25%)
pN1	41 (22%)	46 (24%)
Total	183 (100%)	193 (100%)

**Table 2:** Clinicopathological parameters of the malignant cases

Variable	pT1	pT4	pN1	Total
Number of cases	31 (30.7%)	36 (35.6%)	34 (33.7%)	101 (100%)
Age				
Mean ± SD	60.8 ± 11.8	64.8 ± 16.2	51.71 ± 17.0	59.2 ± 16.1
Range	35–81	15–92	5–83	5–92
Sex				
Female	16 (15.8%)	13 (12.9%)	15 (14.9%)	44 (43.6%)
Male	15 (14.9%)	23 (22.8%)	19 (18.8%)	57 (56.4%)
Breslow’s depth (mm)				
Mean ± SD	0.68 ± 0.23	8.52 ± 9.75		4.89 ± 8.12
Range	0.29–1.00	4.00–60.00		0.29–60.00
Relapse				
Yes	4 (4.0%)	20 (19.8%)	30 (29.7%)	54 (53.5%)
No	26 (25.7%)	14 (13.9%)	1 (1.0%)	41 (40.6%)
Spread at diagnosis	0 (0.0%)	1 (1.0%)	3 (3.0%)	4 (40.6%)
Missing	1 (1.0%)	1 (1.0%)	0 (0.0%)	2 (2.0%)
Anatomic site of primary melanoma				
Head and neck	9 (8.9%)	9 (8.9%)	6 (5.9%)	24 (23.7%)
Trunk	4 (4.0%)	4 (4.0%)	4 (4.0%)	12 (11.9%)
Back	7 (6.9%)	11 (10.9%)	8 (7.9%)	26 (25.7%)
Upper limbs	7 (6.9%)	3 (3.0%)	2 (2.0%)	12 (11.9%)
Lower limbs	4 (4.0%)	3 (3.0%)	10 (9.9%)	17 (16.8%)
Feet	0 (0.0%)	3 (3.0%)	1 (1.0%)	4 (4.0%)
Hands	0 (0.0%)	0 (0.0%)	0 (0.0%)	0 (0.0%)
Fingers or toes	0 (0.0%)	3 (3.0%)	0 (0.0%)	3 (3.0%)
Not found	0 (0.0%)	0 (0.0%)	3 (3.0%)	3 (3.0%)
Cause of death				
Malignant melanoma	1 (1.0%)	19 (18.8%)	26 (25.7%)	46 (45.5%)
Other	7 (6.9%)	8 (7.9%)	0 (0.0%)	15 (14.8%)
Alive	19 (18.8%)	3 (3.0%)	5 (5.0%)	27 (26.7%)
Unknown	4 (4.0%)	6 (5.9%)	3 (3.0%)	13 (12.9%)

### FoxP3 and IDO immunohistochemical stainings

For immunohistochemical stainings, 4 μm thick tissue sections were immunostained separately for FoxP3 and IDO. For FoxP3 stainings, after deparaffinization, the tissue sections were cooked in 10 mM citrate buffer (pH 6.0) in a microwave oven twice for 5 min, and after cooling they were washed with 0.05 M phosphate buffered saline (PBS; pH 7.0). The endogenous peroxidase activity was blocked with 5% H_2_O_2_ for 5 min. Thereafter, the sections were washed and incubated with 1.5% normal horse serum in PBS for 15 min in room temperature to block unspecific binding according to the Vectastain Elite ABC Kit (Vector Laboratories, Burlingame, California, USA). After blocking, the sections were incubated at 4 °C overnight with the primary antibody (1:100, mouse monoclonal anti-Foxp3 antibody, Abcam, Cambridge, UK), followed by incubation with 0.5% biotinylated secondary antibody (1:100, anti-mouse, Vector Laboratories) for 30 min at room temperature. The bound antibody was visualised by using the Vectastain Elite ABC kit (Vector Laboratories, Burlingame, California, USA). The colour was developed with 0.05% 3,3′-diaminobenzidine (DAB) containing 0.8% NiCl and 3% H_2_O_2_ in PBS. Mouse IgG was used as a negative control and tonsil tissue served as a positive control. A counterstain was omitted, because non-specific background staining from the primary immunohistochemical stain was observed to provide adequate anatomical and histological reference to accurately determine the location of the tumor. Finally, the sections were washed, dehydrated, and mounted in DePex.

For IDO stainings, before deparaffinization, the sections were cooked at 58 °C for 30 min. Then, after deparaffinization, the tissue sections were cooked in 10 mM citrate buffer (pH 6.0) in a pressure cooker for 15 min, and after cooling at room temperature, they were washed with 0.1 M phosphate buffer (PB; pH 7.0). Thereafter, the endogenous peroxidase activity was blocked with 1% H_2_O_2_ for 5 min. The sections were then washed and incubated with 1% milk powder in PBS for 30 min at 37 °C to block unspecific binding. The sections were incubated in the primary antibody (1:100, rabbit monoclonal anti-IDO antibody, Cell Signalling, Danvers, MA, USA) at 4 °C overnight, followed by incubation with the biotinylated secondary antibody (1:200, anti-rabbit, Vector Laboratories, Burlingame, CA, USA) for 1 h at room temperature. The bound antibody was visualised with the Vectastain Elite ABC kit similarly to FoxP3 stainings. DAB was used as the chromogen to visualize the stainings, and the nuclei were counterstained with Mayer’s hematoxylin. Negative controls were treated in the same way, but the primary antibodies were omitted. Tonsil tissue was used as a positive control. Thereafter, the sections were washed, dehydrated, and mounted in DePex.

### IDO + CD68, CD68 + CD11c and IDO + FoxP3 immunofluorescence double stainings

Deparaffinized sections were cooked in 10 mM citrate buffer (pH 6.0) in a pressure cooker for 10 min, and they were washed after cooling with 0.1 M phosphate buffer (PB; pH 7.0). Thereafter, the sections were treated with 50 mM glycine for 20 min at room temperature to quench any autofluorescence. The sections were blocked with 1% bovine serum albumin for 30 min, followed by an overnight incubation at 4 °C with the primary antibodies against IDO, CD68 and FoxP3. After washing, the sections were incubated for 1 h with the secondary antibodies (1:400, Texas Red anti-rabbit IgG, Vector and 1:200, Fluorescein-anti rabbit, Vector). Nuclei were labelled with DAPI (1 μg/ml, Sigma-Aldrich). The sections were mounted in Vectashield (Vector H-1000, Vector) and the samples were viewed and imaged with a Zeiss Axio Observer inverted microscope (20 x or 40 x NA 1.3 oil objectives) equipped with a Zeiss LSM 700 confocal module (Carl Zeiss Microimaging GmbH, Jena, Germany).

### Evaluation of Foxp3+ regulatory T cells

FoxP3+ Tregs were evaluated with the hot spot analysis [9–11]. The areas of the highest FoxP3+ cell density (hot spots) were located by scanning each tissue section at low (× 50–100) magnification with a Zeiss Axio Lab.A1 (Carl Zeiss, Germany) light microscope, and then either three or five representative areas (depending on the lesion size) were selected and imaged at × 200 magnification. Tumor cells were present in each picture to ensure proximity to the lesion.

All images were captured using a Zeiss AxioCam ERc 5S microscope-mounted camera (Carl Zeiss, Germany). The images were captured at a resolution of 2560 × 1920 px with a resulting physical pixel size of 0.11 μm/px. From the images, the number of FoxP3+ regulatory T cells was evaluated using automatic digital image analysis (Additional file 1). The results of the automated analysis were verified by two researchers (SS, AL) who independently counted the positive cells manually from 75 randomly chosen samples, so that 25 of these samples were analysed by both investigators. The Pearson correlation for the automated analysis method and manual counts were 0.96 and 0.89, and 0.96 between manual counts. The adequacy of the cell selections completed by the automatic digital image analysis software was also checked for each picture. The cell count was analysed manually in cases where positive cells were significantly over or under selected by the software (271 pictures; 32% of all pictures).

FoxP3+ T cells were distinguished from other FoxP3-expressing cells by the cell size, morphology and staining intensity. Cells were excluded if they were considered too large, had an aspect ratio > 2.0 (ratio of major axis to minor axis), or if they touched the edges of the image. Only cells stained intensely enough to clearly stand out from the background were counted. These criteria were used for manual counting as well as the automated image analysis method.

### Evaluation of IDO stainings

IDO-positivity was evaluated separately from stromal immune cells and melanoma cells. IDO-positive stromal immune cells were counted using the hot spot –method, similar to FoxP3+ regulatory T cells (see the previous section), except that IDO+ stromal immune cell counting in the microscopic images was only done manually. IDO+ stromal immune cells were evaluated based on the nuclear and cell morphology, excluding IDO+ tumor cells and IDO+ endothelial cells or fibroblasts from the analyses. IDO+ stromal immune cells were analysed independently by two researchers (SS, BHG), the Pearson correlation coefficient between the two analyses was 0.88. Samples with significantly distinctive cell numbers were re-evaluated.

The percentage and staining intensity of IDO+ tumor cells were also evaluated from primary melanomas and LNMs. The number of IDO-positive melanoma cells was assessed semi-quantitatively using a 5-level scoring system from 0 to 4. Score 0 was given if the sample contained less than 1% IDO-positive melanoma cells of all tumor cells, score 1 if 1–5% of cancer cells were IDO-positive, and scores 2 to 4 if IDO-positive cancer cells comprised 6–10%, 11–20% and over 20% of all cancer cells, respectively. From those malignant cases that contained > 1% IDO+ tumor cells, the staining intensity of IDO+ melanoma cells was also assessed semi-quantitatively using a 3-level scoring system. Staining intensity was scored 1 for weak, 2 for moderate and 3 for strong. The intensity score was determined by the predominant intensity deposit. IDO+ tumor cells were analysed independently by two investigators (SS, BHG). Samples with a distinct index were re-evaluated by third investigator (SPS). IDO-positive tumor cells were not evaluated from melanin-containing samples because melanin would have interfered with the evaluation.

### Statistical analysis

Statistical analyses were conducted using IBM SPSS Statistics 25 (IBM Corporation, Armonk, New York, USA). A non-parametric Kruskal-Wallis test with pairwise comparisons was used to compare the different histological groups, and a Pearson χ2-test was used to analyze the associations with clinicopathological parameters. A Mann Whitney U-test was used to compare immune cell counts between IDO-positive and IDO-negative tumors. A Kaplan-Meier with log-rank test, and a Cox’s regression were used for univariate and multivariate survival analyses, respectively. For the χ2-test and survival evaluations, the FoxP3+ Treg and IDO+ stromal immune cell counts, analyzed by hotspot analyses, were divided into two groups (low or high) based on the median. Cell counts less than the median value represented low, and cell counts higher than the median represented high cell numbers (median = 56.50 for FoxP3 + Tregs and 40.70 for IDO+ stromal cells). For χ2-tests and survival analyses, the coverage of IDO+ melanoma cells was divided into two groups (IDO-positive and IDO-negative tumors), fusing categories 1–4. Thus, IDO-negative tumors contained less than 1% (category 0) and IDO-positive tumors more than 1% (categories 1–4) IDO+ melanoma cells of all tumor cells. Similarly, IDO intensity in IDO-positive malignant tumors was divided into two groups (either low and moderate or strong) by combining the intensity categories 2 and 3. The higher categories were fused because of small sample sizes. *P* values equal to or less than 0.050 were considered statistically significant.

## Results

### Patient characteristics

Patient and clinicopathological characteristics are presented in Table 2. The mean follow-up was 9.7 ± 8.9 years (median 7.7 years).

### FoxP3+ Tregs and IDO+ stromal immune cells are more abundant in malignant melanoma compared with benign lesions

**Fig. 1:**
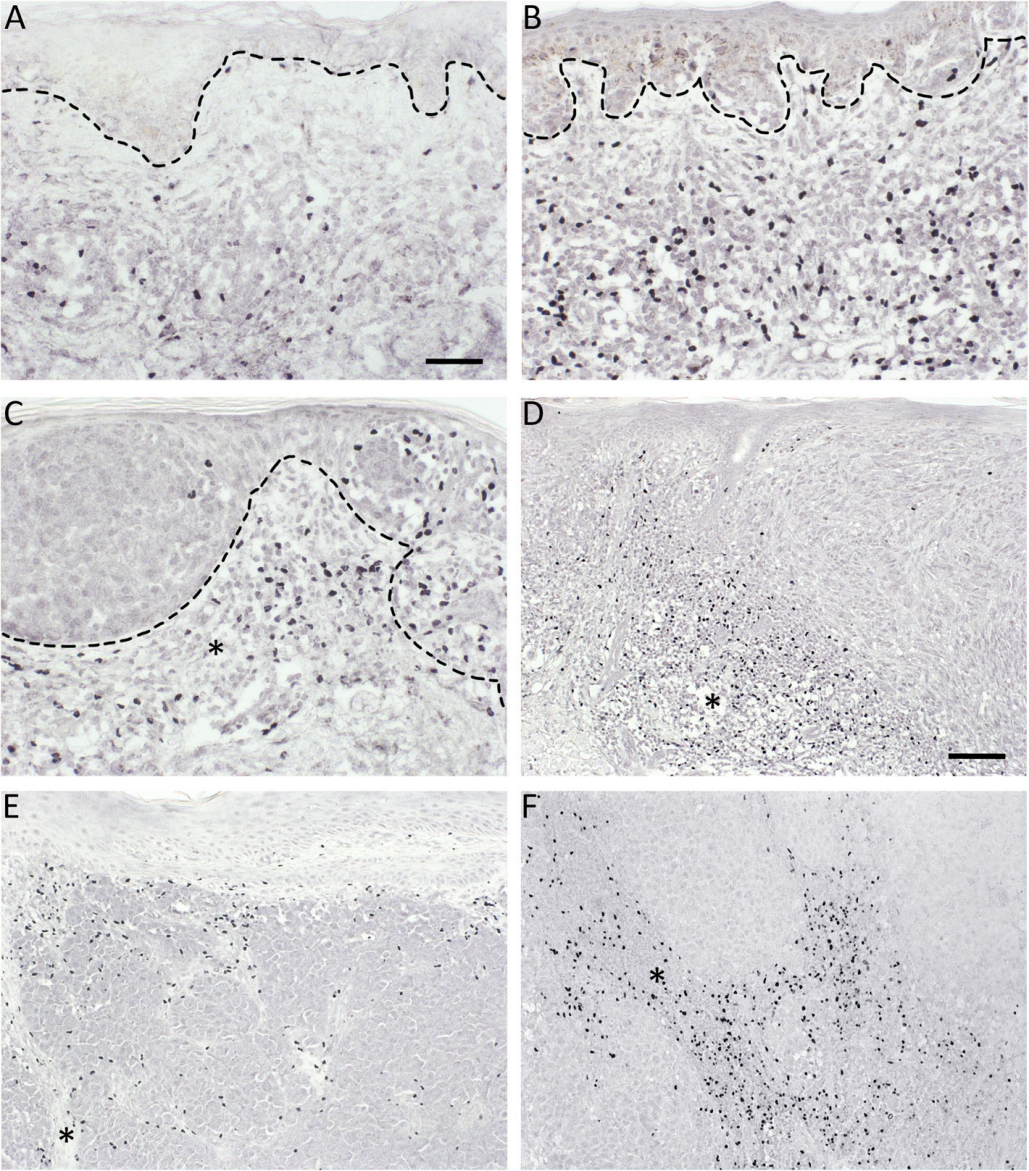
Representative immunohistochemical stainings of FoxP3+ Regulatory T cells (Tregs). Immunohistochemical stainings of FoxP3 in benign (**a**) and dysplastic nevi (**b**), in situ melanoma (**c**), superficial (Breslow’s depth < 1 mm, **d**) and deep (Breslow’s depth > 4 mm, **e**) melanomas and lymph node metastasis (**f**). The dashed line in a and b marks the epidermis in benign and dysplastic nevi, respectively, and the dashed line in c stands for the tumor-stroma borderline in in situ melanoma. In in situ, thin and deep melanomas and LNMs (**c**-**f**), FoxP3+ Tregs are also found inside the tumor cell nests. The asterisks in c-f indicates the stromal compartment of the tumor. Scale bar is 50 μm in a for a-c (× 200 magnification) and 100 μm in d for d-f (×100 magnification)

**Fig. 2:**
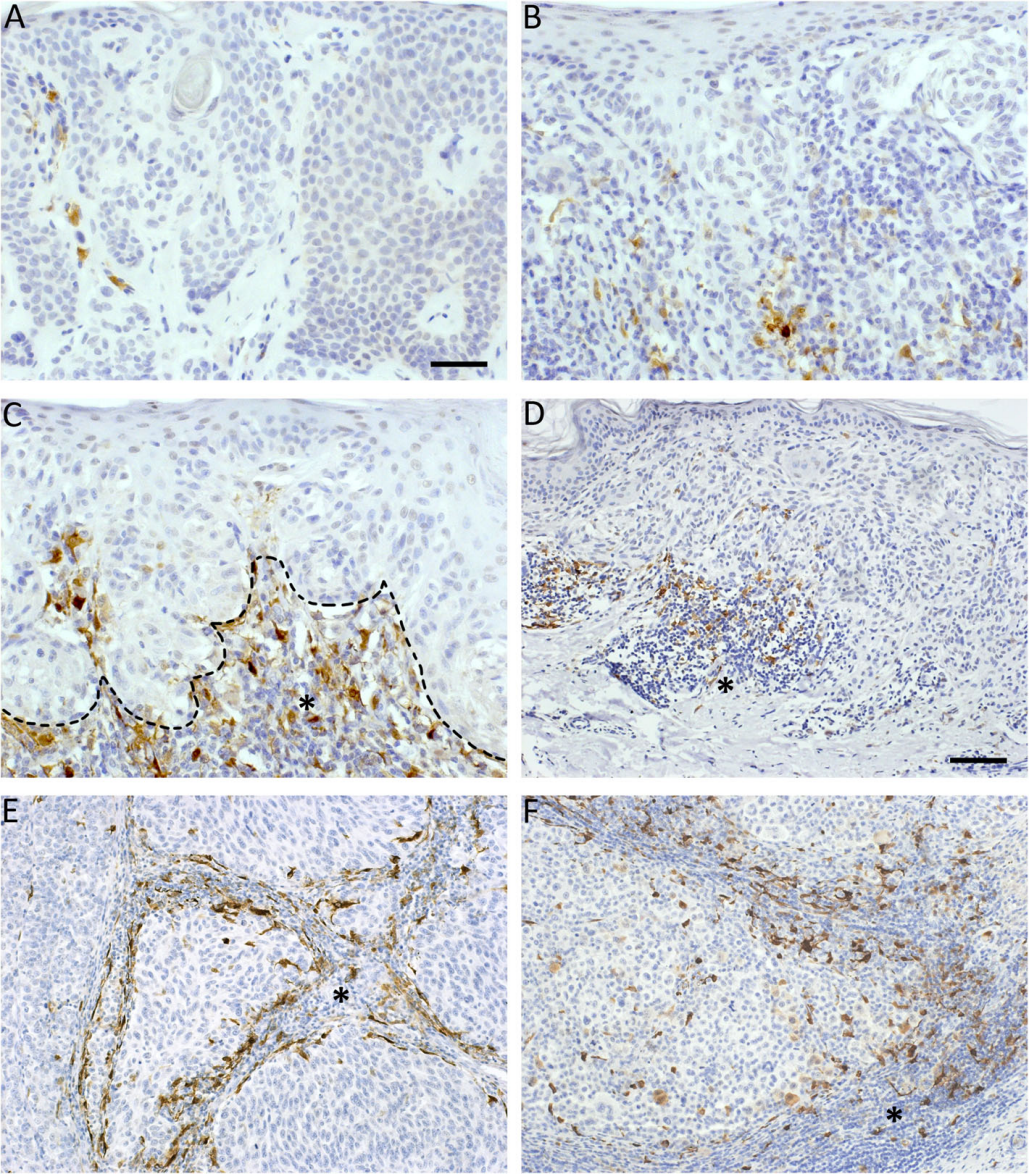
Representative immunohistochemical stainings of IDO. Immunohistochemical stainings of IDO in benign (**a**) and dysplastic nevi (**b**), in situ melanoma (**c**), superficial (Breslow’s depth < 1 mm, **d**) and deep (Breslow’s depth > 4 mm, **e**) melanomas and lymph node metastasis (**f**). The dashed line in c marks for the tumor-stroma borderline in in situ melanoma. IDO+ stromal immune cells were found both intratumorally and in the peritumoral stroma. The asterisks in c-f indicates the stromal compartment of the tumor. Scale bar is 50 μm in a for a-c (× 200 magnification) and 100 μm in d for d-f (× 100 magnification)

**Fig. 3:**
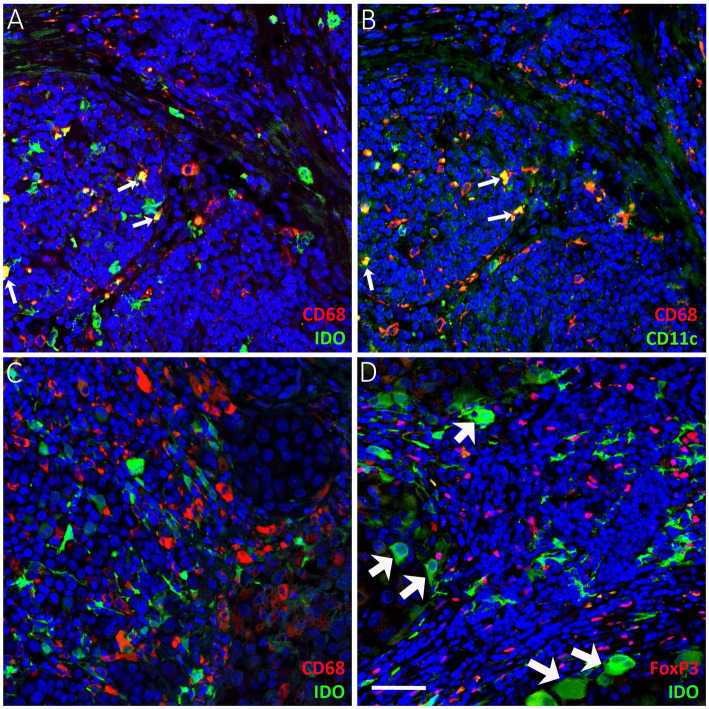
Double immunofluorescence staining of CD68 + IDO, CD68 + CD11c and FoxP3 + IDO. Double stainings for CD68 + IDO (**a**) and CD68 + CD11c (**b**) from serial sections show that part of the CD68+ antigen presenting cells express IDO (a, colocalization, yellow, white arrows) and these same cells are also CD11c + (b, colocalization, yellow, white arrows). Most of the CD68+ cells do not express IDO (**c**), which suggest that the main IDO-expressing antigen presenting cells are CD11c + dendritic cells. Double stainings for FoxP3 + IDO (**d**) shows the colocalization of Tregs with IDO-expressing tumor cells (d, white arrows). a, b and d from lymph node metastases and c from superficial melanoma (pT1). Scale bar 50 μm (× 200 magnification)

Melanoma specimens were stained for FoxP3 and IDO to examine the number and localization of FoxP3+ Tregs and IDO+ stromal immune cells. IDO+ stromal immune cells mainly accumulated in the areas with clear lymphocyte infiltration and thus resided mostly in the perilesional stroma, similar to FoxP3+ Tregs. However, they were also found in intratumoral stroma in deep melanomas and LNMs. The representative immunohistochemical stainings of FoxP3 and IDO are presented in Figs. 1 and 2. Part of the IDO+ stromal immune cells were CD68+ and CD11c + antigen presenting cells (Fig. 3A-B). Double immunofluorescence staining showed that part of the CD68+ macrophages expressed IDO (Fig. 3A-B), and these CD68+ IDO-expressing cells were also positive for the dendritic cell marker CD11c (white arrows in Fig. 3A-B). However, not all CD68+ macrophages were IDO positive (Fig. 3A, C), suggesting that the main IDO expressing antigen presenting cells are dendritic cells. The morphology of IDO+ stromal immune cells also resembled the morphology of dendritic cells with numerous cytoplasmic processes. The staining pattern for IDO was cytoplasmic, while the transcription factor FoxP3 was localized to the cell nuclei (Fig. 3D).

**Fig. 4:**
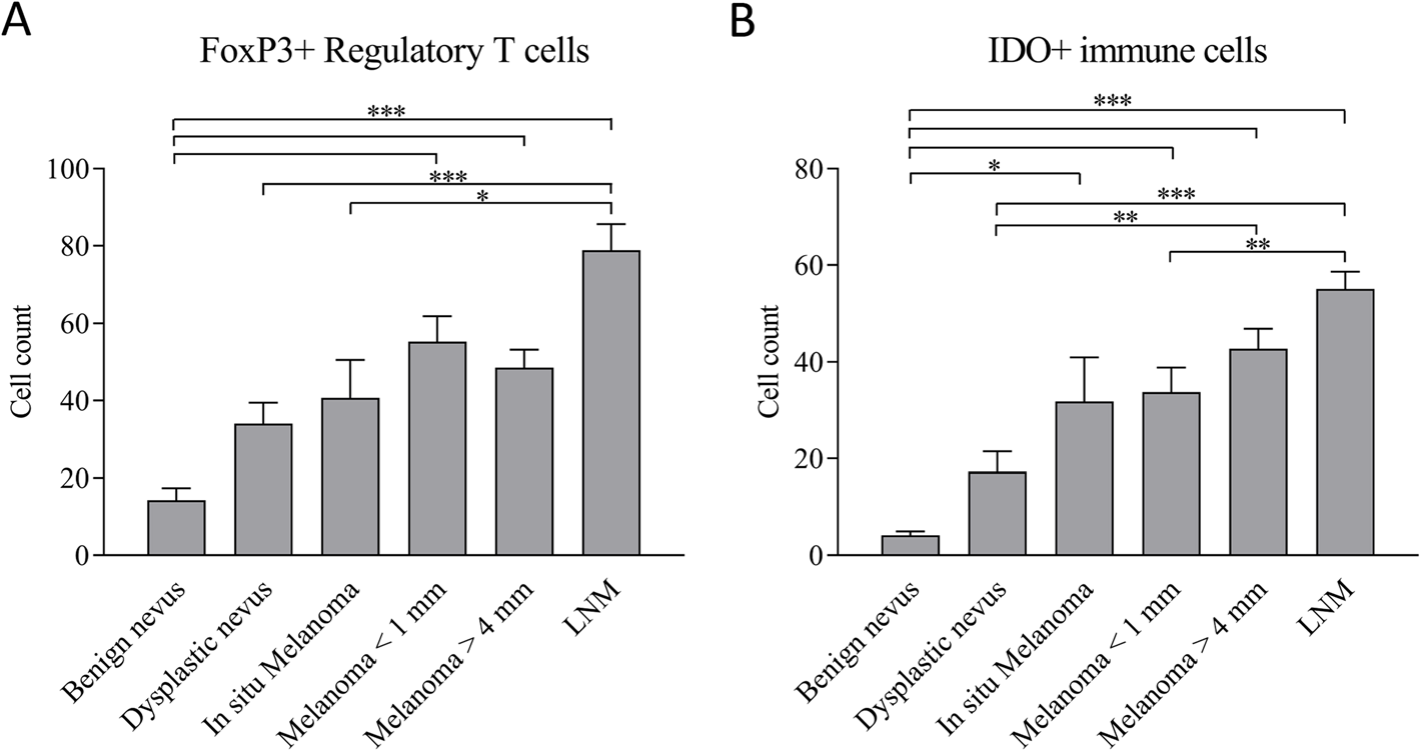
Mean counts of FoxP3+ Tregs (**a**) and IDO+ stromal immune cells (**b**) analyzed by hotspot analysis. FoxP3+ Tregs were analysed from 183, and IDO+ stromal immune cells from 193 melanocytic samples. The data represents mean ± SD. Statistically significant differences between the groups are shown in brackets (Kruskal-Wallis test). *P < 0.05, **P < 0.01, ***P < 0.001

The amount of FoxP3+ Tregs was significantly higher in thin and deep melanomas and lymph node metastases of CM, compared with benign nevi (*p*-values < 0.0001) (Fig. 4A). In line with FoxP3+ Treg counts, the IDO+ stromal immune cell count was also significantly higher in malignant lesions, compared with benign nevi (*p* < 0.0001) (Fig. 4B). The amount of the IDO+ stromal immune cells was higher in deep melanomas and LNMs, compared with dysplastic nevi (p-values 0.003 and < 0.001, respectively), and in in situ melanomas, compared with benign nevi (*p* = 0.012). Interestingly, IDO+ stromal immune cell numbers were also significantly higher in LNMs compared with thin melanomas (*p* = 0.009). However, the correlation between FoxP3+ Treg and IDO+ stromal immune cell counts was rather weak (Pearson’s *r* = 0.43, *P* < 0.001).

### IDO expression in tumor cells is more abundant in malignant melanoma, compared with benign lesions

**Fig. 5:**
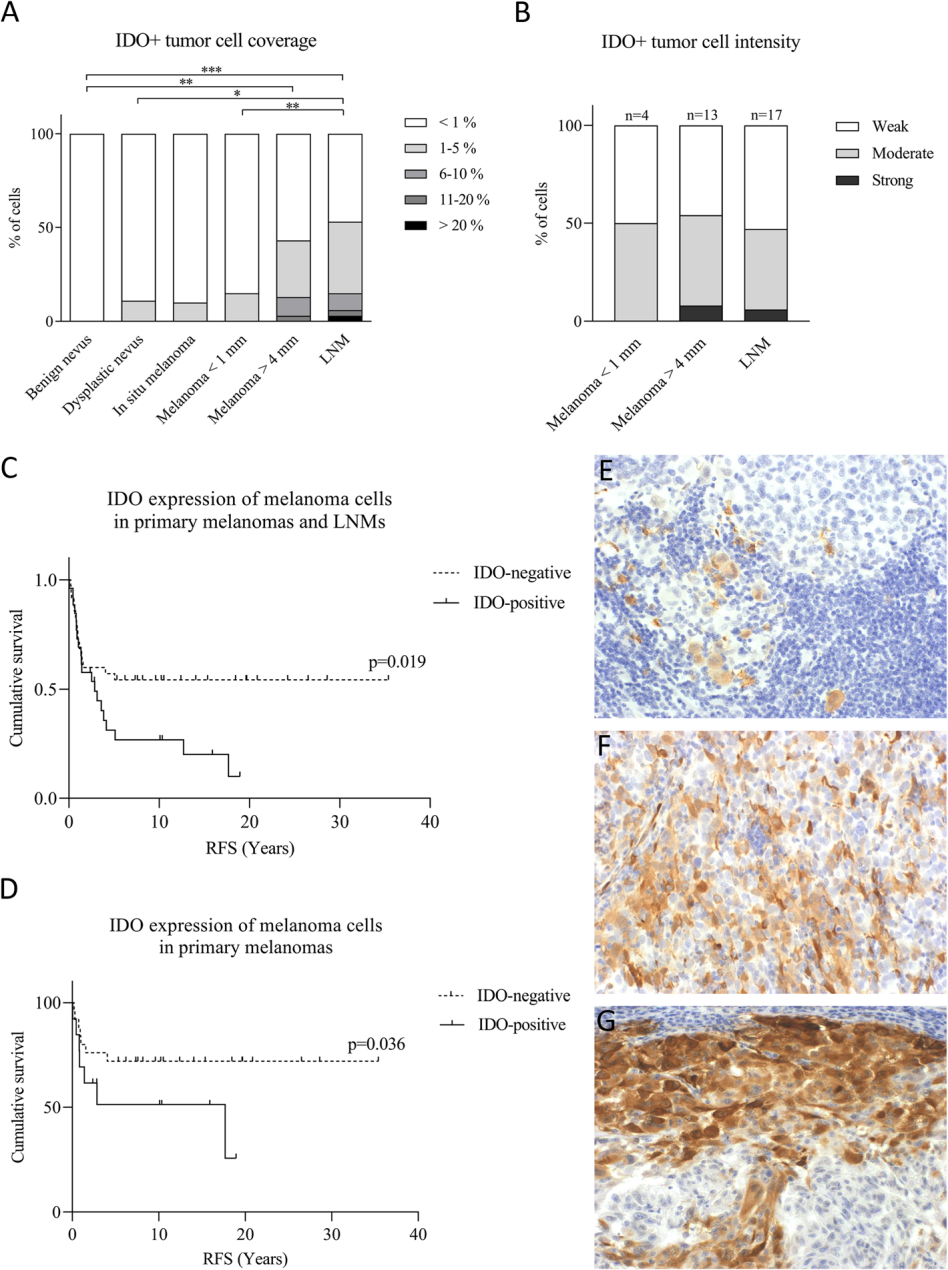
The coverage and intensity of IDO+ tumor cells and their association with survival. IDO+ tumor cell coverage (**a**) was analysed from all samples using 5-level scoring system from 0 to 4. Statistically significant differences between the stages are shown with brackets (Kruskal–Wallistest). *P < 0.05, **P < 0.01, ***P < 0.001. IDO expression intensity (**b**) was also analysed from malignant tumors that contained more than 1% IDO+ tumor cells of all melanoma cells. IDO expression was classified as weak, moderate or strong. Representative stainings for weak, moderate and strong IDO expression in melanoma cells are represented in e-g, respectively. In survival analyses (**c**, **d**) samples were divided into two groups, IDO-negative and IDO-positive tumors, representing tumors that contained less than 1% or more than 1% IDO+ tumor cells of all melanoma cells. IDO-positive tumors was associated with poor recurrence-free survival (RFS) in the group of all samples (pT1, pT4, LNM) (**c**) and in the group of primary melanomas only (**d**) (*P*-values 0.019 and 0.036, respectively)

The coverage and intensity of IDO+ tumor cells was also analysed. The number of IDO+ tumor cells was low, but they most often localized in the invasive front in the same area as the IDO+ stromal immune cells (Fig. 3D). Moreover, IDO+ tumor cells formed IDO-positive islets inside the tumor nests. The staining pattern of IDO+ tumor cells was also cytoplasmic. The coverage of IDO+ tumor cells was significantly higher in deep melanomas and LNMs, compared with benign nevi (*p*-values 0.006 and < 0.001, respectively) (Fig. 5A). Furthermore, IDO+ tumor cells were more abundant in LNMs compared with thin melanomas and dysplastic nevi (p-values 0.009 and 0.014, respectively). There was no statistically significant difference in IDO intensity between malignant tumors that contained IDO-expressing melanoma cells (Fig. 5B).

### Correlations of FoxP3+ Treg and IDO+ stromal immune cell numbers with clinicopathological parameters

High IDO+ stromal immune cell count was associated with the presence of mitoses (*p* = 0.004, data not shown). There were no associations of FoxP3+ Treg numbers with any of the clinicopathological parameters (gender, ulceration, nodular growth pattern, presence of mitoses or recurrence).

### Correlations of IDO expression and intensity of melanoma cells with clinicopathological parameters

To study the association of IDO+ melanoma cell coverage with clinicopathological parameters and survival, samples were divided into two groups; IDO-negative and IDO-positive tumors, which represented samples that contained less than 1% or more than 1% IDO+ tumor cells of all melanoma cells, respectively. The presence of IDO+ melanoma cells was associated with a nodular growth pattern (*p* = 0.050), the presence of mitoses (*p* = 0.047), overall recurrence (*p* = 0.014), as well as locoregional (*p* = 0.009) and distal (*p* = 0.040) recurrence (Table 3). However, the presence of IDO+ melanoma cells was not associated with recurrence when assessing primary tumors only. In addition, moderate or high IDO expression intensity in melanoma cells of LNMs was associated with lymph node capsule rupture (*p* = 0.014).

**Table 3:** Associations of IDO-expressing melanoma cell coverage and intensity with clinicopathological parameters

Variables	IDO expression in melanoma cells			IDO intensity in melanoma cells		
	IDO-negative tumors n (%)	IDO-positive tumors n (%)	*p*-value	Weak n (%)	Moderate or strong n (%)	*p*-value
Growth pattern			**0.050**			1.000
Nodular	11 (52)	10 (48)		5 (50)	5 (50)	
Other	17 (81)	4 (19)		2 (50)	2 (50)	
Presence of mitoses			**0.047**			0.299
Yes	18 (58)	13 (42)		6 (46)	7 (54)	
No	10 (91)	1 (9)		1 (100)	0 (0)	
Lymph node capsule rupture			0.429			**0.014**
Yes	5 (56)	4 (44)		0 (0)	4 (100)	
No	5 (39)	8 (62)		6 (75)	2 (25)	
Overall recurrence			**0.014**			0.352
Yes	16 (44)	20 (56)		11 (55)	9 (45)	
No	19 (76)	6 (24)		2 (33)	4 (67)	
Locoregional recurrence			**0.009**			0.785
Yes	12 (40)	18 (60)		9 (50)	9 (50)	
No	24 (73)	9 (27)		4 (44)	5 (56)	
Distal recurrence			**0.040**			0.472
Yes	16 (46)	19 (54)		10 (53)	9 (47)	
No	20 (71)	8 (29)		3 (38)	5 (63)	

Samples were divided into two groups, IDO-negative and IDO-positive tumors, representing tumors that contained less than 1% or more than 1% of IDO+ tumor cells of all melanoma cells. IDO expression intensity of melanoma cells in IDO-positive tumors was also evaluated, and samples were divided into two groups (weak and moderate or strong expression intensity) for analysis

In univariate survival analyses, IDO-positive tumors were associated with poor recurrence-free survival (RFS), both in analyses conducted in all groups (pT1, pT4, LNMs) (Fig. 5C) and primary melanomas only (Fig. 5D) (*p*-values 0.019 and 0.036, and number on events 36 and 15, respectively). However, the significance was not apparent in multivariate analysis of survival, when the histological group (pT1, pT4 and LNM) or Breslow’s depth was used as a covariate. There was no association of IDO expression in melanoma cells with overall or disease specific survival, and IDO expression intensity did not correlate with survival either.

### IDO-positive tumors contained higher amounts of immune cell infiltrates than IDO-negative tumors

IDO-positive tumors contained significantly higher amounts of FoxP3+ Tregs (*p* < 0.0001), IDO+ stromal immune cells (*p* < 0.0001), and both CD68+ and CD163+ tumor associated macrophages (TAMs) (*p*-values < 0.0001), compared to IDO-negative tumors (data not shown). However, IDO+ stromal immune cell or FoxP3+ Treg counts did not correlate with macrophage counts.

## Discussion

In the present study, our aim was to gain more information on the role and interactions of two important immunosuppressive factors of the TME of CM, FoxP3+ Tregs and IDO. We show that high amounts of IDO-expressing melanoma cells correlate with poor prognostic factors and poor RFS in CM. Moreover, the number of both FoxP3+ regulatory T cells and IDO+ stromal immune cells was shown to be more abundant in malignant melanomas, compared to benign lesions. We also analysed the tumor immune cell infiltrates in relation to IDO-expressing melanoma cells and found that IDO-positive tumors contain significantly higher amounts of FoxP3+ Tregs, IDO+ stromal immune cells, and both CD68+ and CD163+ tumor associated macrophages (TAMs), compared with IDO-negative tumors. It has been shown that an immunosuppressive TME is a driving factor for tumor growth and disease progression in CM [12]. Our study of two immunosuppressive factors, FoxP3+ Tregs and IDO, support this notion. In particular, IDO expression in the melanoma cells seem to play an important role in melanoma progression, and our results also suggest that FoxP3+ Tregs and IDO+ immune stromal cells enhance tumorigenesis in CM.

Our results support earlier reports on the association of IDO+ stromal immune and tumor cells with poor prognosis in CM. For example, Rubel et al. found that high amounts of both IDO+ melanoma and stromal cells correlate with poor progression-free survival (PFS), and high amounts of IDO+ melanoma cells were also positively associated with Breslow’s depth [13]. In another study, abundance of IDO-expressing melanoma cells in LNMs of CM was associated with poor OS [14]. Similarly, our results indicate that IDO expression in both stromal immune cells and tumor cells promote tumorigenesis in CM, as the number of both IDO+ stromal immune cells and IDO+ tumor cells was significantly higher in malignant compared with benign lesions. In addition, overexpression of IDO in melanoma cells was associated with poor prognostic factors and recurrence. Importantly, IDO has already been studied as a target of immunomodulatory treatments in CM. Preliminary results from clinical trials assessing the effects of IDO-inhibition to patient survival rates in CM have been promising [5].

However, previous studies related to FoxP3+ Tregs in CM have been contradictory. In some studies, high amounts of FoxP3+ Tregs have been correlated with greater Breslow’s depth or poor survival. For example, Gerber et al. reported that high amounts of FoxP3+ Tregs was associated with reduced OS but there was no correlation with Breslow’s depth [15]. Miracco et al. found that a high percentage of FoxP3 + CD25 + Tregs correlated with recurrence in vertical growth phase melanoma [16]. While in other studies, a correlation between FoxP3+ Tregs and tumor stage or prognosis in CM has not been found [17–19]. Surprisingly, one study also indicated that high amounts of FoxP3+ Tregs was associated with better prognosis in CM [20]. Tjin et al. evaluated several immune escape markers and immunosuppressive cells in stage IV melanomas; they reported very low expression of Foxp3+ in tumor-infiltrating immune cells, which suggested a minor role for Tregs in stage IV melanomas [21]. Our study also indicates that FoxP3+ Tregs are not as strongly involved in CM tumorigenesis as IDO+ cells. We found that malignant melanocytic lesions contained higher amounts of FoxP3+ Tregs compared with benign nevi, but FoxP3+ Tregs was not associated with clinicopathological parameters, tumor stage or survival.

Discrepancies between studies in the the role of FoxP3 + Tregs in CM may be partly due to the different study materials and/or cell counting methods used. Furthermore, FoxP3 is not expressed exclusively by Tregs; indeed, some conventional CD4+ T cells with no immunosuppressive properties also express FoxP3, as well as some CD8+ T cells, referred to as regulatory CD8+ T lymphocytes [22]. Thus, a more accurate assessment of the role of Tregs in CM could be made using CD25 co-staining [23]. Only a few retrospective immunohistochemical studies have assessed CD25 + FoxP3+ Tregs in CM. One study found CD25 + FoxP3+ Tregs was positively associated with recurrence in vertical growth phase melanomas [16], whereas another study found CD25 + FoxP3+ Treg counts in tumor-stroma boundary were higher in atypical nevi and radial growth phase melanomas, compared with vertical growth phase melanomas and melanoma metastases [24].

It is worth noting that FoxP3+ Tregs consist of distinct tissue-specific subsets that have diverse phenotypes and functions, and presumably they are differentiated and activated by tissue-specific cues, and regulated by various transcription factors [25]. Thus, it is possible that different tumors with specific TMEs modify the phenotype and function of tumor-infiltrating Tregs. This would also at least partly explain why FoxP3+ Tregs seem to promote tumor-progression in some cancers, such as colorectal cancer [26–28], while inhibiting tumor growth in other cancer types, such as breast cancer [29]. In addition, Treg diversity may also partially explain why Tregs have a controversial role in some other malignancies, including gastric cancer [30], and it may also contribute to the discrepancies between immunohistochemical studies on the role of FoxP3+ Tregs in CM.

In the present study, IDO expression in tumor cells was positively associated with high amounts of FoxP3+ Tregs. The same result was obtained in another study, where the study material consisted of primary melanomas, LNMs and distal metastases of CM [31]. Similarly, Brody et al. reported that upregulation of IDO in tumor cells of LNMs was associated with an increased number of Tregs in CM, as well as with shorter survival [32]. However, the correlation between IDO+ stromal immune cell and FoxP3+ Treg counts was surprisingly weak. To our knowledge, no study has assessed or reported a correlation between Foxp3+ Tregs and IDO+ stromal immune cells in human CM. Thus, we can only speculate that the activation and stabilization of Tregs may not be the primary function of IDO+ stromal immune cells in CM. Furthermore, IDO is a counter-regulatory molecule, whereby its expression is induced by inflammation or T cell activation [33]. Thus, the association of IDO+ melanoma cells with high amounts of FoxP3+ Tregs and TAMs may not refer to the direct impact of IDO on Tregs and TAMs, but to a high overall immunological activity and strongly induced immune suppression in the tumor site.

There are some limitations that should be taken into account when interpreting the present results. Only pT1 and pT4 melanomas were included in this study, as these tumors have clearly distinct prognosis. Thus, we did not study melanomas > 1 mm or < 4 mm in our study. In addition, the tissue samples stained for FoxP3 and IDO were not taken from serial sections; however, this factor is not seen to notably affect the results.

In summary, our results indicate that IDO expression is intimately involved in creating a TME conducive to tumor growth and disease progression in CM. Furthermore, we show that FoxP3+ Tregs appear to contribute to the immunosuppressive TME in CM, but their role may not be that critical to melanoma progression. Additional research is still needed to assess the role of Tregs in the context of different TMEs, and to clarify the interactions between IDO expressing tumor cells versus IDO+ stromal immune cells with Tregs in CM.

## Conclusions

Our study supports the existing perception that IDO plays a pro-tumoral role in CM. We found that the number of IDO+ stromal immune cells is higher in malignant melanomas, compared with benign melanocytic lesions, and that higher amounts of IDO+ melanoma cells correlate with poor prognostic factors and poor RFS in CM. We also studied the association between IDO and FoxP3+ Tregs, which has not been previously addressed substantially in CM, and we found a positive correlation between IDO+ melanoma cells and FoxP3+ Tregs. Despite our finding that FoxP3+ Treg numbers were higher in malignant melanomas, compared with benign melanocytic lesions, we did not find a correlation between IDO+ stromal immune cells and FoxP3+ Tregs. This latter result may indicate complex interactions between IDO and Tregs in CM, which demands further studies.

## Supplementary Information

**Additional file 1: Figure 1.** Simplified flow chart of the computer vision algorithm used for automated image analysis. **Figure 2.** Melanin rejection filter Input (left) and output (right). **Figure 3.** Cell detection results. Input image (left), visualisation of algorithm output (middle) and output visualisation overlaid on top of input image (right).

## Data Availability

The datasets supporting the conclusions of this article are available on request from the corresponding author (satu.salmi@uef.fi).

## Abbreviations

CM: Cutaneous Melanoma
DC: Dendritic cell
DSS: Disease-specific survival
FoxP3: Forkhead box P3-transcription factor
IDO: Indoleamine-2,3-dioxygenase
LNM: Lymph node metastasis
MDSC: Myeloid-derived suppressor cell
NK: Natural killer cell
OS: Overall survival
PBS: Phosphate buffered saline
RFS: Recurrence-free survival
TAM: Tumor associated macrophage
TME: Tumor microenvironment
Treg: Regulatory T cell
