# Supplementary material for: The role of FoxP3+ regulatory T cells and IDO+ immune and tumor cells in malignant melanoma – an immunohistochemical study
Source: BMC Cancer. 2021 May 29;21:641. doi: 10.1186/s12885-021-08385-4 (PMC8164759; doi:10.1186/s12885-021-08385-4)
Supplement: Supplementary file 1 — Additional file 1: Figure 1. Simplified flow chart of the computer vision algorithm used for automated image analysis. Figure 2. Melanin rejection filter Input (left) and output (right). Figure 3. Cell detection results. Input image (left), visualisation of algorithm output (middle) and output visualisation overlaid on top of input image (right). [file 12885_2021_8385_MOESM1_ESM.pdf]

Supplementary Material 1.

# Automated image analysis used in FoxP3+ Regulatory T cell evaluation

## 1 Outline of the image analysis method

FoxP3+ Regulatory T cells were counted using a semi-automated computer vision program. The program uses a combination of image processing methods to identify and quantify FoxP3 positive cells in the input image (Fig. 1). Special care was taken to minimise the number of false positives with filters designed to reject FoxP3-expressing non-lymphocyte cells.

### 1.1 A brief description of the image analysis algorithm

The input image is first color vector filtered to selectively reject melanin containing areas while preserving the darker FoxP3 stain (Fig. 2). A grayscale image is then obtained by computing the minimum decomposition ($Value_{x,y} = \min(R_{x,y}, G_{x,y}, B_{x,y})$) of the color filtered image. Adaptive Otsu thresholding (6x6 sampling grid, 10% sample overlap) is performed to produce a (1-bit) mask of positively stained areas. The mask is then median filtered (5px radius) to reduce high frequency noise that could adversely affect subsequent processing steps and object detection. Contiguous areas in the mask are then labelled and sorted by size. Areas of appropriate size for a single lymphocyte are passed on to the cell feature filter stack. Areas larger than a single lymphocyte (presumed to be artefacts or clusters of cells) are segmented using a size-selective marker-based watershed transform and then passed through the cell feature filter.

### 1.2 The cell feature filter stack

The cell feature filter stack consists of consecutive filter stages designed to mimic the criteria used in manual counting. The filter stages work independently and reject cells that do not meet the criteria. For the purposes of this study, filters for size (min/max), roundness (isoperimeter quotient), aspect ratio, intensity relative to the background, intensity relative to other cells and proximity to image edges were used.

## 2 Validation of the image analysis method

Results from the automated image analysis were validated against a set of images counted independently by the authors. The output of the automated analysis was generally found to correlate very well with the manually counted set. Distinguishing artefacts and FoxP3-expressing tumour cells from FoxP3 positive lymphocytes proved to be a challenge, but good false-positive rejection rates were achieved with careful selection and tuning of filters. A further challenge was posed by the varying cell and background staining intensity between slides. The aforementioned factors were found to account for most of the errors in the automated analysis. However, with well selected image processing methods and carefully tuned settings a good level of accuracy was achieved. All results of the digital analysis were confirmed by the authors and corrections to the cell counts were made as needed.

## 3 Benefits and drawbacks of automated digital image analysis

One of the major benefits of digital image analysis in comparison to traditional counting methods is the vastly superior speed at which the analysis can be carried out. This enables the processing of much larger sets of data in a given amount of time. As a result, a greater number of samples can be analysed in more detail, as the sampling density and area can be increased. Digital image analysis algorithms are also objective by nature and produce consistent and repeatable results.

Drawbacks of digital image analysis include the difficult and time-intensive nature of developing and setting up an automated image analysis pipeline with currently available tools. Machine learning could be used to automate parts of the calibration and set up process and is a possible topic for further research.

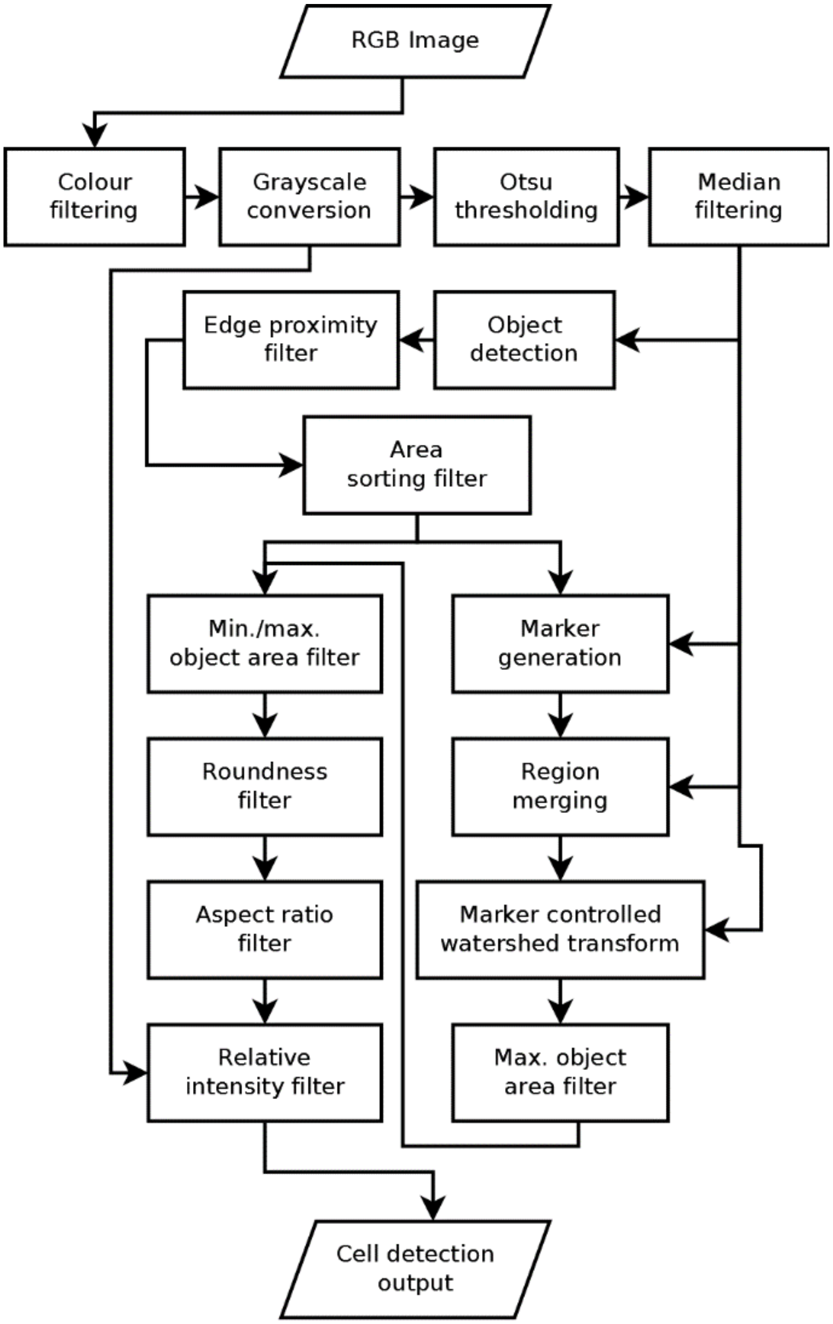


**Figure 1.** Simplified flow chart of the computer vision algorithm used for automated image analysis.

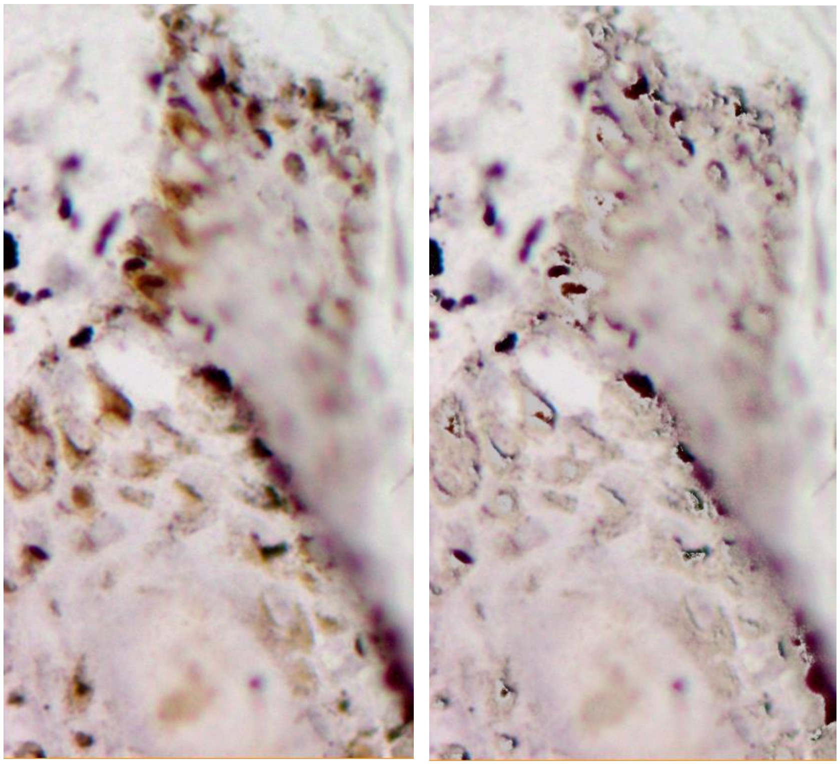

**Figure 2.** Melanin rejection filter Input (left) and output (right).

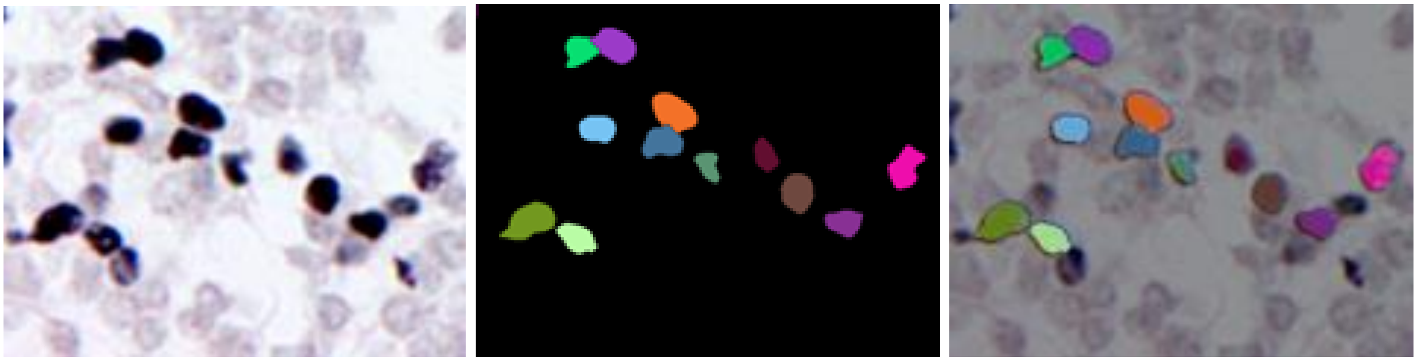

**Figure 3.** Cell detection results. Input image (left), visualisation of algorithm output (middle) and output visualisation overlaid on top of input image (right).
